# A theory-based multicomponent intervention to reduce occupational sedentary behaviour in professional male workers: protocol for a cluster randomised crossover pilot feasibility study

**DOI:** 10.1186/s40814-020-00716-9

**Published:** 2020-11-10

**Authors:** Gail Helena Nicolson, Catherine Hayes, Catherine Darker

**Affiliations:** Public Health & Primary Care, Trinity College Dublin, Institute of Population Health, Russell Centre, Tallaght Cross, Dublin, D24 DH74 Ireland

**Keywords:** Under-desk pedal machine, mHealth, Sedentary behaviour, Active sitting, Physical activity, Occupational sedentary behaviour, Socio-ecological model

## Abstract

**Background:**

Prolonged sitting, a significant risk factor for increased morbidity and mortality, is accumulated mostly in the workplace. There is limited research targeting specific at-risk populations to reduce occupational sedentary behaviour. A recent study found that professional males have the longest workplace sitting times. Current evidence supports the use of multi-level interventions developed using participative approaches. This study’s primary aims are to test the viability of a future definitive intervention trial using a randomised pilot study, with secondary aims to explore the acceptability and feasibility of a multicomponent intervention to reduce workplace sitting.

**Methods:**

Two professional companies in Dublin, Ireland, will take part in a cluster randomised crossover pilot study. Office-based males will be recruited and randomised to the control or the intervention arms. The components of the intervention target multiple levels of influence including individual determinants (via mHealth technology to support behaviour change techniques), the physical work environment (via provision of an under-desk pedal machine), and the organisational structures and culture (via management consultation and recruitment to the study). The outcomes measured are recruitment and retention, minutes spent sedentary, and physical activity behaviours, work engagement, and acceptability and feasibility of the workplace intervention.

**Discussion:**

This study will establish the acceptability and feasibility of a workplace intervention which aims to reduce workplace SB and increase PA. It will identify key methodological and implementation issues that need to be addressed prior to assessing the effectiveness of this intervention in a definitive cluster randomised controlled trial.

**Supplementary Information:**

The online version contains supplementary material available at 10.1186/s40814-020-00716-9.

## Introduction

### Background and rationale

Prolonged periods of daily sedentary behaviour (SB) are associated with increased mortality, cardiovascular morbidity, diabetes [1–3], some cancers [4, 5], depression [6], and decreased self-rated health [7]. SB has been defined as any waking behaviour while in a sitting or lying position that expends ≤ 1.5 metabolic equivalents (METs) of energy expenditure (EE) [8]. Being sedentary for more than 7 h per day is associated with increased all-cause and cardiovascular mortality rates [3, 9]. Although high levels of physical activity (PA) may attenuate these relationships, 60–75 min per day of moderate physical activity (MPA), or 3.5 times the World Health Organization’s (WHO) PA recommendations of 150 min of MPA per week, are required to eliminate the detrimental effects of SB [9]. Time spent in SB has increased rapidly in middle- to high-income countries in recent years and is set to continue to do so without intervention [10]. Given the detrimental health impact of prolonged and uninterrupted daily SB, this presents a serious public health concern.

The settings approach moves interventions upstream from defining goals and targets in terms of populations or individuals only, towards identifying goals that focus on changes in organisations, systems, and the environment [11, 12]. In this context, all of the opportunities for influencing health within a setting can be considered priorities for change, which can lead to maximised health gain [13]. The WHO includes the implementation of multicomponent interventions in a workplace setting to target physical inactivity as one of their ‘best buy’ recommendations for the prevention and control of non-communicable diseases [14].

Working adults spend more than 7.5 h of their day being sedentary, and when individual, social, and environmental factors are controlled for, professional males with high levels of education and who live in an urban location have the longest sitting times [15–17]. High levels of occupational SB are associated with depression and anxiety [18], increased BMI [19], and risk of heart failure [20], mortality in men [21], colorectal cancer [22], pancreatic cancer [23], lung cancer in women [23], and breast cancer in women aged younger than 55 years [24]. Reducing workplace SB is important to curtail the physical and mental health risks associated with prolonged SB [6, 25–27]. Individuals in private offices sit more and engage in more prolonged sitting than those in public office spaces suggesting that this group may be at increased risk of the associated health outcomes [28].

Given the strong reinforcing and restrictive properties of the physical and social environment of the office workplace, allowing workers to continue with their favoured or required task (e.g. computer work), while breaking up prolonged SB may be most acceptable and effective in terms of workplace interventions to address SB [29]. Studies examining sit-stand workstations have enabled the break-up of prolonged SB by replacing some SB with standing; however, standing does not provide the metabolic benefits of light physical activity (LPA) [30]. A recent study employing a data compositional approach showed that standing (and SB) was associated with increased body mass index (BMI), body fat, and fat mass [31]. Standing for long periods may indeed be detrimental to cardiovascular health and has been associated with an increase in the risk of ischemic heart disease and varicose veins [32]. Rempel and Krause [33] suggest that advising sedentary employees to increase standing time at work should not be recommended, and maintain that if the basis for a reduction in SB is to improve cardiovascular health, promotion of increased standing is misguided. Results from studies using treadmill desks [34] and activity-permissive workstations [35] suggest that combining simultaneous, low-intensity PA with sedentary practices could increase daily caloric expenditure and reduce cardio-metabolic risk factors. ‘Active sitting’ as opposed to ‘reduced sitting’ may be preferred in workplace interventions where the choice of employees and/or employers may be to remain seated [36]. Furthermore, combining PA with sedentary activities could reduce time-related costs of PA—a frequently cited barrier to regular PA in adults [37].

The socio-ecological model of SB emphasises the importance of intervening at the many levels influencing behaviour in the workplace and includes organisational structures, the physical and social interpersonal environment, and intrapersonal factors [38]. Multicomponent interventions have been most successful in reducing workplace SB [39], while interventions that involve environmental restructuring (e.g. activity-permissive workstations) have shown the largest reductions in daily SB [40]. Cycling workstations with resistance (i.e. 20–30 Watts) can increase EE by twice the amount of METs compared with standing workstations [41]. The feasibility of using under-desk portable pedal machines to reduce SB has been reported in laboratory settings [42, 43] and in studies predominantly of women [44]. Some productivity issues while cycling in work, i.e. accuracy of computer mouse dexterity [45] or typing performance [43], have been reported; however, cycling does not impair reading comprehension or speed of computer mouse use. These productivity issues were inversely related to cycling speed, whereby cycling at a high cadence is likely to result in considerable trunk movement, providing a less stable base for upper limb movements and hence potentially impairing the task performance [43, 45]. Cycling in work has been found to increase arousal and reduce boredom significantly more than standing workstations [46] and may be capable of increasing short-term memory and attention more effectively than standing or treadmill workstations [47]. The rationale for providing sedentary employees working in professional environments with pedal machines at work is to allow participants to engage in light-intensity activity (i.e. active sitting) that can be performed for long periods throughout the day without causing perspiration, a previously reported barrier to workplace PA [48, 49].

Ensuring relevance and individualisation are effective basic methods in health interventions, and this is traced especially to social cognitive theory (SCT) [50]. The core determinants of health behaviour in SCT are knowledge of health risks, perceived self-efficacy, outcome expectations, health goals, perceived facilitators and barriers, and social and structural impediments to change. In a recent review of behaviour change techniques (BCTs) used in SB reduction interventions among adults, the most frequently used intervention functions were enablement (i.e. facilitating reduction in SB), education, and environmental restructuring. The most commonly used (and also most promising) techniques were setting behavioural goals, providing unspecified forms of social support, and addition of objects to the environment [51].

Contemporary technological advances in digital tools such as mobile phones, the Internet, and wearable technology provide a platform to intervene on an individual level to change behaviours. A systematic review and meta-analysis of interventions using computer, mobile, and wearable technology to reduce SB reported a mean reduction of 41 min per day in interventions that used these tools. The most frequently used BCTs were prompts/cues, self-monitoring of behaviour, unspecified social support, and goal-setting [52].

Mobile health (mHealth) technology has rapidly gained popularity in the general population. mHealth technology includes wearable PA monitors and trackers that connect to smartphone applications (apps). These apps allow individuals to manage their own health and wellbeing at a relatively low cost and offer potential to tailor interventions to the needs of individuals or specific groups. A recent review to investigate the use of mHealth in interventions found reasonable evidence that mHealth may be an effective and feasible method to increase PA, with some evidence for effectiveness in reducing SB [53]. Studies using mHealth to promote PA and reduce SB in the workplace found significant reductions in sedentary time in women [54], where the outcomes were increasing daily steps [55, 56], or reductions in computer use as a proxy for SB [57]. Team-based competition as opposed to individual monitoring has been found to increase compliance with wearing activity monitors [53].

Adopting a participatory approach to intervention development and evaluation of an intervention’s acceptability and feasibility benefits the development of effective interventions [58, 59]. Pretesting the participants’ knowledge, beliefs, and circumstances and using this information as a basis for intervention development creates relevance [60]. For interventions to be acceptable, feasible, and effective, participant involvement provides important information on the individual, organisational, and cultural contexts into which SB reduction strategies must be embedded.

### The present study

This study operationalises the Eldridge et al. [61] definition of a randomised pilot study, i.e. those studies in which the future RCT, or parts of it, including the randomisation of participants is conducted on a smaller scale. Feasibility outcomes which ‘might be interviews to ascertain the acceptability of an intervention’ are also investigated within Eldridge et al.’s feasibility study description [61].

Previous research attests to the potential efficacy of combining pedal machines and motivational behaviour change strategies. However, to our knowledge, no studies have combined BCTs of goal-setting, social comparison, self-monitoring, and prompt/cues in a multicomponent intervention using mHealth technology, an ergonomic under-desk portable pedal machine, as well as targeting organisational support by recruiting management staff to participate, in a male-only sample. Pilot work can aid in the design of future trials with continuous outcomes by providing estimates of population SD, evidence of potential for intervention effectiveness, and quantification of feasibility in the form of recruitment and retention rates [62]. This paper outlines the protocol for a randomised pilot study which will employ a cluster randomised controlled wait-list crossover design.

#### Aims and objectives

The study aims to:

1. To conduct a randomised pilot study to test a set of feasibility objectives to ascertain if a future RCT is viable
2. To investigate the acceptability and feasibility of a multicomponent intervention to reduce SB by promoting active sitting and LPA in professional male office workers

The objectives of this study will test the feasibility and viability of the intervention in a future larger trial by reporting recruitment and retention rates, and potential intervention effectiveness by ascertaining if participants in the intervention period differ in occupational and overall SB and PA (including cycling time) compared to the control period. This will be addressed by collecting accelerometry data, which will provide information on minutes spent sedentary, standing and moving. The objectives also include an investigation of the acceptability of the intervention using semi-structured interviews and focus group data which will explore participants’ views of acceptability and usefulness and their expectations and experiences of the study. The study will measure intervention acceptability, appropriateness, and feasibility using a questionnaire administered to participants immediately post-intervention [63].

## Methods

### Design

#### Intervention development process

This intervention was developed using guidance from the Medical Research Council (MRC) [64, 65] and encompasses three distinct phases. The first preclinical stage was a literature review of workplace interventions to reduce SB and the application of socio-ecological theory to the design of the current intervention.

Phase I involved the identification of intervention components and the underlying mechanisms by which the outcomes will be influenced. The development of the intervention followed the principles of the integrated approach of socio-ecological theory: a method that emphasises the need to consider multiple levels of influence on behaviour. Qualitative testing and the adoption of a participatory approach through focus groups and semi-structured interviews with both employees and managers have been conducted to help understand the relevance of the intervention components as well as potential barriers to behaviour change.

This protocol outlines phase II of the approach that tests the acceptability and feasibility of an intervention to reduce workplace SB to develop an optimum intervention, as well as feasibility objectives to test the viability of a future trial. The dominance of progression criteria has been noted as relatively crude and somewhat binary assessments of acceptability and feasibility (e.g. [53]). Assessing the acceptability and feasibility of complex interventions in terms of what works, for whom and under what circumstances, and aiming to refine hypotheses about potential mechanisms of action and how these might vary by context has been suggested as more appropriate to better develop interventions [66] and can be integrated to the MRC framework [65, 67]. By exploring the views of those involved by collecting rich qualitative data, as well as contextual exploration, enables optimisation of intervention design or how to adapt to different contexts prior to a full RCT. Piloting the processes of a full RCT such as randomisation and assessing qualitative data provides insight without biasing outcome measurement, e.g. Hawthorne effect, and can assist in hypothesis refinement.

The intervention comprises the provision of the following components: (1) an under-desk portable pedal machine (DeskCycle2; 3D Innovations LLC., Greeley, CO, USA), (2) Garmin Forerunner 35 activity tracker, and (3) access to a Garmin Connect application (app) and website (Garmin.com). The organisational-level component will be targeted by recruiting management staff to the study, thereby garnering support for employees to participate in the pilot study.

The intervention will communicate the key message: ‘cycle at work’. As highlighted from prior qualitative work, the determinants goal-setting, self-monitoring, and social comparison will be included using BCTs provided within the Garmin Connect app/website. Gender-sensitised interventions that recognise men’s interests and tailor health promotion efforts for this specific group have been found to be more effective in increasing physical activity [68, 69]. Social comparison will be used as a strategy to focus on the masculine ideal. By engaging men in PA, this draws upon as well as provides opportunities to garner masculine capital by affirming competitiveness and/or striving for physical prowess [68, 70]. Social comparison will be targeted by providing weekly feedback of each team’s progress. Participants will also be prompted to move every 60 min of accumulated SB using the ‘move’ prompt on the Garmin Forerunner 35 wrist-worn device.

**Fig. 1:**
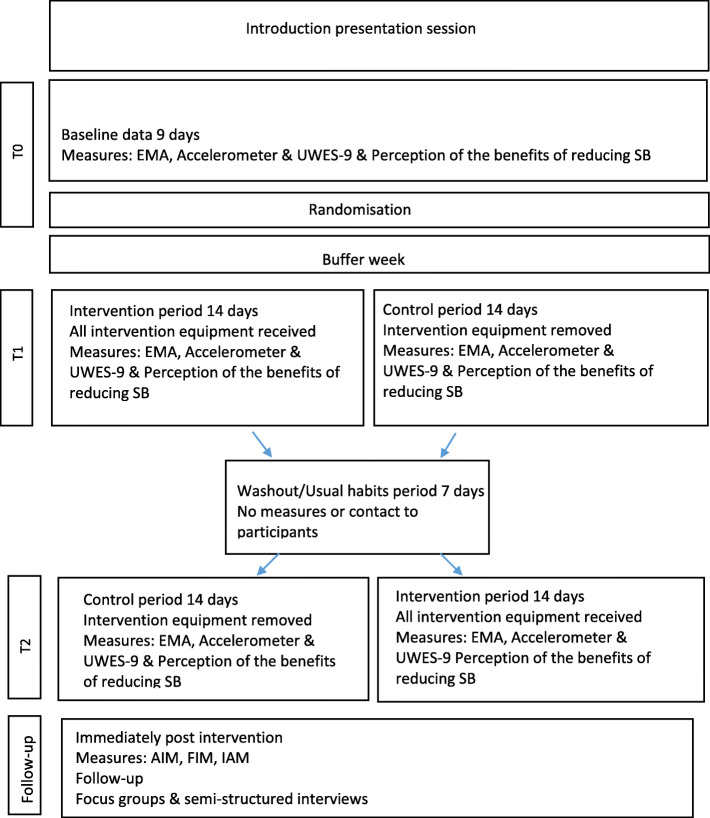
Flow chart of the ‘cycle at work’ study

Figure 1 shows an overview of the study timeline. The intervention and control arms will be conducted over 14 days each. The active intervention will involve the use of an under-desk pedal machine to interrupt SB every hour and accumulate ≥ 30 min of cycling time during the working day. There will be a washout period of 1 week between the intervention and control arms. The washout period is used as there is a possibility that the effect of a treatment/intervention in one period may carry over into the next period [71]. These are known as carryover effects. Unless both carryover and period effects are known to be negligible, a crossover design loses its advantages. In order to ensure negligible carryover effects, there is a need to have sufficient washout periods between intervention periods. In circumstances where a participant suffers any adverse outcome such as pain or discomfort while taking part in the study, they will be advised to immediately discontinue participation in the study and to contact their doctor.

The study protocol has been registered at the International Standard Randomised Controlled Trial Number (ISRCTN11584275).

The Standard Protocol Items: Recommendations for Intervention Trials reporting guidelines were used to guide the preparation of this pilot study protocol [72].

#### Participants

Participants will be office-based employees from two professional companies in Dublin, Ireland.

##### Inclusion criteria

- Males who spend most of their working week performing desk-related activities

##### Exclusion criteria

- Have limitations with or contraindications to physical activity as indicated by the Physical Activity Readiness Questionnaire [73]
- Do not have a personal desk
- Females
- Age under 18 years
- Employees who plan to be absent from the workplace for more than 2 days during the study period
- Employees who are involved in another programme or intervention to reduce behaviours

#### Randomisation

Following baseline assessments, worksites will be randomised to the intervention or control arms of the trial. Simple cluster randomisation will be determined by a statistician not associated with the project, who will use randomisation software to allocate each worksite to begin with either the intervention or control period.

#### Allocation concealment

Participants will not be advised of their group allocation until after baseline assessments have been made. The allocation concealment mechanism is important to reduce selection bias as it prevents foreknowledge of the period (control/intervention) in which participants are enrolling, which negatively affect recruitment [74].

#### Blinding

Due to the nature of the study, neither the participants nor the research team will be blinded to group assignments.

#### Setting and context

The proposed randomised pilot feasibility study will be conducted in two private-sector professional organisations in Dublin, Ireland (a legal firm and an online medical education provider). The sites have been chosen to target professional males as outlined in the introduction as those with the highest risk of prolonged SB.

Management approval has been obtained for employee recruitment, for permission to make environmental changes in the office setting, and for study contacts to occur during work-time. All participants will provide written informed consent before inclusion in the study.

#### Selection and recruitment

Convenience sampling has been used to recruit the organisations who have been involved in the development of this pilot study. The organisations were initially approached through the lead researcher’s personal networks. Purposive sampling will be used to recruit eligible participants in the companies via an email sent by a contact within each company. Participants in this study will include members of management and managing partners as well as employees.

#### Sample size

No formal sample size calculations are produced for this pilot feasibility study. The sample size is pragmatic and chosen based on resources. Thirty male desk-based workers will be recruited to the feasibility trial. A sample size of *n* = 30 is conventionally deemed adequate for pilot studies as it permits collection of sufficient useful data while minimising research costs [75].

Focus groups comprising separate management and employee participants in each worksite will be recruited (four in total) for intervention evaluation purposes. This is appropriate in qualitative research of this kind, with diversity of sampling (i.e. all stakeholder groups) more important than numbers of focus groups [76].

#### Procedure

An open call will be given to all staff who meet inclusion criteria, regardless of area/department to take part in the research. When preliminary agreement to the study has been obtained, the lead researcher will meet potential participants at their workplace, where they will be provided with a consent form, participant information leaflet (PIL), and a verbal explanation of the study. Participants who are interested in taking part in the study will be asked to consider the consent form and PIL for a 24-h period. Arrangements to meet all participants who are willing to participate will then be made, and they will then sign the consent form. The Physical Activity Readiness Questionnaire (rPARQ) health screening tool [73] will be administered to participants at the information/briefing stage to ensure participants’ physical capability to safely participate in the study.

Following the baseline period, all participants will be provided with a report via email on their weekly SB and PA derived from their baseline accelerometer data. Participants randomised to the intervention group will then be shown by the lead researcher in a face-to-face session at their workplace on the correct use of the intervention equipment. The goal of cycling 30–40 min/day (i.e. ≥ 5 min/h for 8 h) has been chosen as the minimum amount of exercise break to fractionate SB [31]. The Garmin watch is paired with a Bluetooth cadence sensor on the pedal machine and will record minutes of cycling upon start and stop buttons pressed by participants. The completed activity will transfer wirelessly via Bluetooth to a smartphone application (Garmin Connect) or to the website on participants’ workplace computer using a wire. The watch has a ‘move bar’ that visually appears and provides a sound and vibration alert after 1 h of inactivity. Additional segments appear every 15 min of inactivity thereafter. The move bar is reset by engaging in a small amount of physical activity (i.e. work short distance, record stationary cycling). The Garmin platform does not allow the setting of SB goals, but does allow cycling-time goals. Prior to the intervention commencement, all participants will be assigned teams formed within sites (e.g. managers versus employees, or a mix of roles), which will target the social comparison behaviour change strategy. The Garmin platform allows self-monitoring of participants’ own time spent in SB, PA, and cycling, and participants will be encouraged to visit the site regularly. A weekly email from the researcher will provide encouragement and feedback on participants’ activity progress. For logistical and practical reasons, there will be a buffer week after randomisation. This is to allow the researcher to attend the workplaces to deliver the pedal machines and Garmin watches.

#### Control arm

Participants in the control arm will be informed that they have been randomised to a delayed intervention that will begin after 3 weeks and will be asked to continue their normal workplace habits. All measures collected in the intervention group will be collected in the control arm.

#### Assessments

At baseline, participants will wear the thigh-based accelerometer (activPAL3) monitor for 24 h/day, for nine consecutive days (and 14 days each for control and intervention arms). All device removals will be documented in a wear diary. Prior to being attached to the participant, the device will be set to record at 20 Hz. The activPAL3 will be set to start recording at 0001 h on the day after the participant receives the device. Each device will be attached to the anterior aspect of the midline of the right thigh using a nitrile sleeve and waterproof Tegaderm dressing. Sleep, sedentary time, standing time, physical activity (i.e. stepping time (minutes) (cadence ≥ 100, duration > 1 min) will be derived from the activPAL3 data. An acceleration threshold has been developed (unpublished data) to identify under-desk cycling, i.e. cut-point threshold acceleration exceeding 375.0 (sum of vector magnitude), while seated (recorded as SB by activPAL), and in bouts lasting ≥ 5 continuous minutes. Only cycling that occurs within self-reported working hours will be analysed and then quality-checked by comparing to user-entered Garmin recorded cycling time. Sedentary time and standing time will be calculated using the postural function of the monitor through the associated software (activPAL v8.10.8.75).

Contextual SB information will be measured using self-report via Ecological Momentary Assessment (EMA). The use of EMA has been recommended to collect ecologically valid and context-specific outcome data alongside objective measures in studies [77, 78]. EMA involves repeated sampling of participants’ current behaviours and experiences in real time and in their natural environments. This is useful to specify the type of activity or contextual factors (e.g., physical, social, temporal, affective) surrounding these behaviours which are important factors to consider when developing interventions and that cannot be provided by objective measures [79]. EMA has been reported as a valid and reliable measure of SB and PA in adults [80] and for use in a workplace setting [81]. Each day, six notifications will appear on participants’ own mobile smartphones at random times between 8 am and 10 pm, using the application PIEL Survey (pielsurvey.org, v1.2.4.2). The notifications are scheduled at random times to obtain a representative sample of participants’ activities over the course of their study participation. The questions have been found to be valid and feasible [82].

Work engagement will be measured at baseline, post-control arm, and post-intervention arm using the Utrecht Work Engagement Scale (UWES-9) [83]. The perceived benefits of reducing SB in the workplace will also be assessed at these time points using a questionnaire devised by the research team. Immediately upon finishing the study, participants will be asked to complete a questionnaire to assess the acceptability, appropriateness, and feasibility of the intervention [63].

Focus groups and semi-structured interviews will be carried out within the 2-week post-intervention follow-up. An interview schedule has been designed based on existing literature. The interview schedule for the focus groups will be guided by Orsmond et al. [84]. The schedule will be pre-piloted on a small number of employees within the author’s place of work, within the Discipline of Public Health and Primary Care.

#### Pilot outcomes

Trial-related outcomes will be explored within the focus groups and/or semi-structured interviews which includes:

- Acceptability of the assessments and burden by the users—from a management and employee perspective
- Acceptability of the study procedures by the users—from a management and employee perspective

##### Recruitment and retention

- Number of people recruited to the study recorded by the researcher at the beginning of the study
- Number of dropouts in the study will be recorded

##### Feasibility of measurement tools

- Missing data from questionnaires. This information will be recorded by the researcher in a separate report at the end of the study.

##### Potential intervention effectiveness

Trial-related outcomes will be assessed at baseline (before randomisation) and throughout the control and intervention periods:

- SB and PA measured using ActivPal3 accelerometers:

-SB in minutes during working hours (workplace SB) and all waking hours (total SB)

-PA in minutes during working hours (workplace PA) and all waking hours (total PA)

- Context-specific SB measured using EMA with notifications of survey completion sent six times a day at random times throughout the baseline, control, and intervention arms
- Work engagement will be measured at baseline, post-control arm, and post-intervention arm using the UWES-9 [85] using pen and paper
- Perception of the benefits of reducing workplace SB will be assessed using the 3-point questionnaire at baseline and immediately post-intervention using pen and paper

#### Feasibility outcomes

The following quantitative measures will be used [63]:

- Intervention appropriateness measure (IAM)
- Acceptability of the intervention measure (AIM)
- Feasibility of the intervention measure (FIM)

Evaluation of participants’ perspectives of the intervention will be assessed via focus groups and/or semi-structured interviews using the following themes:

- Experience of using the under-desk pedal machines, including factors perceived as affecting the pedal machine, issues (e.g. contextual, practical, individual, or others), and adverse consequences (e.g. work, health, or otherwise related)
- Experiences of the mHealth intervention components (e.g. Garmin watch)
- Organisational-level and management perspectives on using the pedal machine
- Acceptability of the overall intervention by the users, from a management and employee perspective

#### Progression criteria

As pilot studies are usually too small to estimate parameters required for estimating a sample size for a main cluster randomised trial (e.g. the intra-cluster correlation coefficient) with sufficient precision, and too small to provide reliable estimates of rates for process measures such as recruitment or follow-up rates, these are not calculated in the present study [86]. This study is an exploratory study and progression criteria should not be judged as strict thresholds but as guidelines using, for example, a traffic light system with varying levels of acceptability [86, 87]. We will decide whether or not to proceed to a fully powered RCT using the following assessment principles and progression criteria:

- Green: indicates that we have met a criterion or we are within 10% of our stated progression targets
- Amber: indicates that we are within 20% of our stated progression target, in which case we will critically review reasons for this and assess whether major changes to study methods are likely to realise significant improvements
- Red: indicates that we are more than 20% from our target, in which case we will not, in the absence of clear extenuating circumstances, consider progression to a full trial

Progression criteria include protocol non-adherence and outcome data.

##### Protocol adherence criterion

Green—≥ 80% of participants engage in > 60% of their cycling goal

Amber—60–79% of participants engage in > 60% of their cycling goal

Red—<60% of participants engage in > 60% of their cycling goal

##### Retention progression criterion

Green—≥ 80% of participants provide main trial-related outcomes (SB/PA) at T2

Amber—60–79% of participants provide main trial-related outcomes at T2

Red—< 60% of participants provide main trial-related outcomes at T2

### Data analysis

Descriptive analysis will account for the recruitment and retention. Quantitative analyses will be carried out using Statistical Package for the Social Sciences V.25 (IBM Corp., Armonk, New York, USA). Descriptive statistics (e.g. daily mean SB and PA in minutes, SD) will be provided for all questionnaire data, from the EMA information, and overall SB and PA as derived from the objective measure.

Participant experience of acceptability and satisfaction with the intervention, as well as trial-related processes, will be assessed using analysis of the focus groups and semi-structured interviews. Transcriptions of audio-recorded interviews will be analysed using thematic analysis [88]. At each stage, findings will be verified and discussed in order to assess the accuracy of the interpretation, promote reliability, and ensure rigour [89]. The main analysis of this study will include thematic analysis, and no software package will be used to analyse the data.

## Discussion

This paper describes the design of a cluster randomised wait-list crossover pilot feasibility trial that will test pilot outcomes which will ascertain if a future larger-scale RCT is viable. Acceptability and feasibility outcomes of this theory-led multicomponent intervention to reduce SB in professional males will also be discussed. The design builds on previous developmental work in the participating worksites. The current study, to our knowledge, will be the first study to target professional males using an intervention that combines an under-desk pedal machine; the utilisation of mHealth to target specific BCTs such as self-monitoring, social comparison, and goal-setting; and recruitment management staff to the study. This unique combination of components aims to reduce SB and increase LPA during participants’ working day. The design of the study has been underpinned by the socio-ecological theory acknowledging that an understanding and subsequent targeting of the intrapersonal, interpersonal, physical environmental, and cultural-level factors are likely to be required to achieve the greatest changes in behaviour [38].

Office workers are one of the largest occupational groups in high-income countries and are sedentary for a large proportion of their day; therefore, reducing their SB could have important public health implications by reducing this risk factor associated with chronic disease and mortality [15]. Reallocating just 30 min of SB, sleep time, or standing time with LPA has been found to beneficially affect body composition, including BMI and fat mass [31]; therefore, restructuring the physical environment to enable LPA is an important strategy.

The current randomised pilot feasibility study is designed to inform subsequent refinement of intervention content, in terms of acceptability and feasibility of the intervention components and measures, so that the format may be suitable for real-world implementation and evaluation in a future definitive trial. Its primary purpose is to address key design uncertainties, including the feasibility of recruiting eligible participants, as well as the appropriateness, acceptability, and feasibility of the intervention. The qualitative component of the study will allow for exploration of any issues surrounding the acceptability of the under-desk pedal machines, as well as the mHealth component from the perspectives of the users, which will include employees and management. It will also allow for exploration of the study procedures and assessment methods.

By assessing the potential effectiveness outcomes of SB and PA, and work engagement, and the perceived benefits of reducing workplace SB, the current pilot feasibility study will clarify the design of a future larger trial that will extend the current knowledge regarding the effectiveness of this type of multicomponent intervention to reduce occupational SB.

## Supplementary Information

**Additional file 1:.** SPIRIT 2013 Checklist: Recommended items to address in a clinical trial protocol and related documents.

**Additional file 2:.** Ecological momentary assessment.

**Additional file 3:.** Focus group schedule.

**Additional file 4:.** Participant Information leaflet and consent form.

**Additional file 5:.**

## Data Availability

Not applicable—protocol paper.

## Abbreviations

SB: Sedentary behaviour
METs: Metabolic equivalents
EE: Energy expenditure
PA: Physical activity
MPA: Moderate physical activity
WHO: World Health Organization
LPA: Light physical activity
BMI: Body mass index
SCT: Social cognitive theory
BCT: Behaviour change technique
MRC: Medical Research Council
rPARQ: Physical Activity Readiness Questionnaire
EMA: Ecological Momentary Assessment
UWES-9: Utrecht Work Engagement Scale
IAM: Intervention appropriateness measurement
AIM: Acceptability of intervention measurement
FIM: Feasibility of intervention measurement
