# Supplementary material for: A theory-based multicomponent intervention to reduce occupational sedentary behaviour in professional male workers: protocol for a cluster randomised crossover pilot feasibility study
Source: Pilot Feasibility Stud. 2020 Nov 10;6:175. doi: 10.1186/s40814-020-00716-9 (PMC7653741; doi:10.1186/s40814-020-00716-9)
Supplement: Supplementary file 2 — Additional file 2:. Ecological momentary assessment. [file 40814_2020_716_MOESM2_ESM.docx]

## Appendix 2 Ecological momentary assessment

1. What were you doing right before the phone went off?

Response options: Reading, Using computer, watching TV/movies, Eating/drinking, Socialising, Doing hobbies, Physical activity/exercising, Other

1. What type of physical activity/exercise?

Response options: Running/jogging, Walking, Weightlifting/strength training, Using cardiovascular equipment, Elliptical machine, Cycling, Other

1. What was this other activity?

Response options: Cooking/chores, Riding in a car, Childcare, Attending meeting/appointment, Volunteering, Something else

1. Were you sedentary while doing that activity?

Response option: Yes, No
