# Supplementary material for: A theory-based multicomponent intervention to reduce occupational sedentary behaviour in professional male workers: protocol for a cluster randomised crossover pilot feasibility study
Source: Pilot Feasibility Stud. 2020 Nov 10;6:175. doi: 10.1186/s40814-020-00716-9 (PMC7653741; doi:10.1186/s40814-020-00716-9)
Supplement: Supplementary file 3 — Additional file 3:. Focus group schedule. [file 40814_2020_716_MOESM3_ESM.docx]

## Appendix 3 Focus group schedule

1. Acceptability of intervention components

To start, could you tell me what are your thoughts of the intervention, overall? *What was your experience of it?*

What did you think of the individual components of the intervention? *What was your experience of using the pedal machine? How did you find wearing the Garmin watch? What about the challenge aspect of the intervention?*

What are your thoughts on the measures used within the study? *How did you find wearing the accelerometer?*

What are your thoughts on the text messages asking about your physical activity and sedentary behaviour? *The number of messages throughout the day – too many? Was it acceptable to you? Were there any other outcomes of using this that you were not expecting?*

1. Randomisation

What are your thoughts on the fact that you were in the control group or the intervention group to begin the study? *How might it have affected your participation in the study?*

1. Appropriateness of the intervention

Do you think that the intervention was appropriate in helping you to be less sedentary and move more in your working day? *Do you think the components used in the intervention were appropriate in enabling you to reduce your workplace sedentary behaviour?*

1. Effectiveness of the intervention

Do you think that the intervention was effective in its aims of reducing your sedentary behaviour and increasing your physical activity in work? *Do you feel that the intervention achieved its goal in reducing your workplace sedentary behaviour? Do you think this is sustainable in the long-term?*

1. Barriers to reducing workplace sitting not addressed by the intervention

Was there something that impeded you taking part in the intervention that we did not take into consideration? *Was there a factor that stopped you taking part in the intervention as much as you would have liked?*

1. Other benefits/harms of the intervention

Were there other benefits or improvements that you felt because of the intervention?

Were there disadvantages taking part in the intervention?

1. Suggested improvements to the intervention

What do you think would improve the intervention going forward if it was run on a larger scale? *What would you change if you were to take part again in the intervention? Would you stick to it over a long period?*
