# Supplementary material for: A theory-based multicomponent intervention to reduce occupational sedentary behaviour in professional male workers: protocol for a cluster randomised crossover pilot feasibility study
Source: Pilot Feasibility Stud. 2020 Nov 10;6:175. doi: 10.1186/s40814-020-00716-9 (PMC7653741; doi:10.1186/s40814-020-00716-9)
Supplement: Supplementary file 4 — Additional file 4:. Participant Information leaflet and consent form. [file 40814_2020_716_MOESM4_ESM.docx]

## Appendix 4 Participant Information leaflet and consent form

**
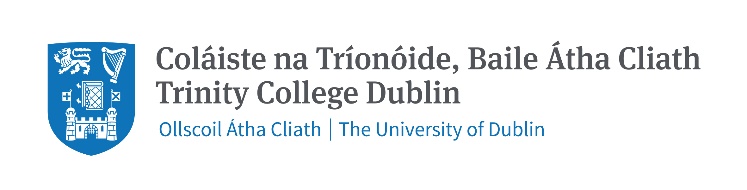
**

“Pilot study to test the acceptability and feasibility of a theory-led multicomponent intervention to reduce sedentary behaviour in the workplace”

**Research Team**

Gail Nicolson, Dr Catherine Darker and Dr Catherine Hayes

We are inviting you to take part in a research study. Before you decide that you want to take part, it is important for you to understand why it is being done and what it will involve. Please take you time to read the information in this information sheet before deciding to take part. If you have any questions or do not understand the information, you can ask the research team. Their details are at the end of this information sheet.

**What is the aim of this research?**

The aim of the research is to undertake a pilot study to investigate if a multicomponent intervention to reduce sedentary behaviour in a workplace setting is acceptable and feasible.

**Who is organising the research?**

The study is being conducted by Gail Nicolson as part of her PhD project to test a pilot intervention to reduce sedentary behaviour in the workplace. The PhD is funded by the Dean of the Faculty of Health Sciences, Trinity College Dublin.

**Can I take part in this study?**

We are looking for men aged 18 or over with sedentary occupations, who are physically healthy to engage in light-moderate physical activity, and who would like to reduce their sedentary in their working day.

**How many people will take part in the research?**

Thirty people will take part in this pilot study.

**What are the possible risks to taking part in the study?**

There are minimal risks to taking part in this study.

**What are the possible benefits of taking part in this study?**

Participants in this study are contributing to the understanding of the acceptability and feasibility of an intervention to reduce sedentary behaviour in a workplace setting. People taking part will potentially reduce their daily sedentary behaviour and may thereby benefit from taking part in the study.

**Do I have to take part?**

You do not have to take part in this study. You may decide if you would like to take part. You are free to refuse to take part in the pilot study, refuse to answer have any measurements taken at any time. You are free to withdraw from the study at any time and your details will be deleted if you decide to withdraw or request that your information is deleted.

**What will happen if I take part?**

If you decide to take part in the research, you must sign a consent form. We would like to see if an intervention to reduce sedentary behaviour at your workplace is acceptable and feasible, and also if it is effective in reducing your sedentary behaviour and increasing your physical activity.

Firstly, to get your baseline daily activity information we will ask you to wear a thigh-worn accelerometer to measure your sedentary behaviour and physical activity for 24 hours a day for 7 days. From this information we will provide you of a graph illustrating a breakdown of your sedentary behaviour and physical activity for the week. We will also ask you to download an app that will notify you 6 times a day every day to complete a short survey (each survey takes approximately 10 to 30 seconds to complete) to help us to gain real-time information about what you are doing throughout the day. The questions ask what you are doing right before the notification went off – such as if you are working on your computer, reading or engaging in physical activity.

Your worksite will then be randomised to start the study either in the control period which means that you will not receive the intervention but you will continue to wear the accelerometer data and you will be sent the text messages asking about your daily activities. At the end of each week the researcher will come to your workplace to upload the accelerometer data onto a laptop. Alternatively, your worksite will begin with the intervention period, followed by the control period depending on the randomisation. The control periods and intervention periods will take place over 14 days each. In between the control and intervention periods will be what is called a ‘washout period/usual habits’ for 7 days where you have no measurements taken and you will not be contacted by the researcher.

In the intervention period you will receive an under-desk pedal machine (Desk-Cycle^TM^) to use as well as a wrist-worn physical activity tracker (e.g. Garmin Forerunner 35) so that you can track your daily use of the pedal machine and monitor your activity using the associated app/website (e.g. Garmin Connect). You will take part in a challenge to cycle at your desk every day and upload your activity to the website where you can see yours-, and others in your worksites’ progress. The activity tracker will also prompt you to move every hour that you have been sedentary, and by engaging in some physical activity such as a short walk or uploading a cycling activity, you will clear this ‘move bar’.

In summary, there is a 7 day baseline measure period, then you will then either be in the intervention period or the control period (14 days each); with a 7 day washout period in between; followed by whichever period you did not receive. At the end of the baseline, control and intervention periods we will ask you to complete a questionnaire on your work engagement. This questionnaire takes approximately 5-10 minutes to complete.

At the end of the study, we would like you know how you found the study and what your experience and thoughts of participating were. We would like you to complete a short questionnaire (takes about 5 minutes to complete) on whether you thought that the intervention was acceptable, feasible and appropriate. We would also like you to take part in a focus group to tell us about your experience of being in the study. We will convene in a place suitable to you to carry out the focus group. The focus group will consist of 6-8 of your fellow co-workers to discuss your views on the pilot study in your workplace. The discussion will be audio-recorded and will take 30 - 40 minutes. The recording will be sent to a transcriber who will put it into writing word for word. Your name or any identifying information will not be included in the transcript and the audio recording will then be destroyed. Your information will not be disclosed to anyone outside of the research team. You can request a transcript of the interview.

**What will happen to the information that I provide?**

We will keep all of your information confidential. Your name and contact details will only be seen by the research team.

The information (data) collected from the accelerometer will be uploaded to a secure laptop that is password encrypted. The accelerometer device only collects data on your activities such as lying, sitting, standing, stepping and cycling in minutes per day. Your name will not be attached to any of this data and each information file will be have a code when uploaded.

The PIEL Survey app is only used to collect the survey data and send you notifications. The PIEL Survey app does not use a remote server or database. Your data is stored on your own phone. At the end of each study period you will email your data file to the researcher. The email account on your devices is set up to use SSL/TLS security using settings from the email provider ensuring encryption.

The questionnaires used in this study will be completed using pen and paper and your name will not be attached to them. All completed questionnaires will be stored in a locked bag during transportation to the researcher’s place of work, where they will be stored in a locked cabinet.

We will replace your name with a code and store your name separately from your other information. Only the researcher will hold the key to the code. The researcher will enter the information that you provide on a password-protected computer using the code. The data will then be analysed by the researcher. Trinity College Dublin is the Data Controller. The means that the College controls and is responsible for the keeping and use of your personal information. The transcriber of the focus group data is the Data Processor - that is they process your data. They must only process your data on the instructions of the Data Controller. The responsibilities of the Data Processor include the necessity to keep personal data secure from unauthorised access, disclosure, destruction or accidental loss. The Data Processor will destroy the audio recording when it is transcribed. Your data will not be used in future unconnected research without your consent. If you would like to have more information about how your data are protected please ask for the Privacy Notice. You can ask for a copy of the Privacy Notice from Gail Nicolson (details below). In line with Trinity College Dublin Data Protection guidelines all data will be stored securely for ten years. Your information will be destroyed securely after that time.

If you need to make a complaint, you can contact the Data Protection Officer at [dataprotection@tcd.ie](mailto:dataprotection@tcd.ie).

**What will happen to the study results?**

The results of the study will be used in the write-up of the researcher’s PhD thesis. Research results may also be published in a journal or presented at a conference. Your information will not be linked to you in any way.

**Has this study been approved?**

The study will not begin until approval is received from Research Ethics Committee of the School of Medicine at Trinity College Dublin.

**Further information:** If you would like any further information, or have questions about the study and your participation in the focus group, you can contact Gail Nicolson on 01-8963739.

**
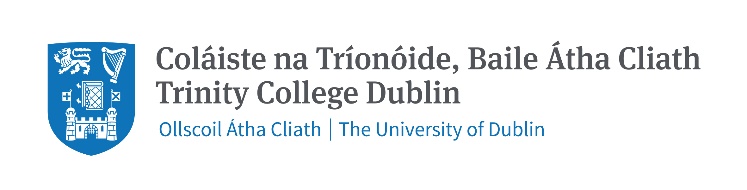
**

**Interview Participant Consent Form**

Pilot study to test the acceptability and feasibility of a theory-led multicomponent intervention to reduce sedentary behaviour in the workplace

**PhD Candidate**: Gail Nicolson, Trinity College Dublin.

Tel: 01-8963739 Email: [nicolsg@tcd.ie](mailto:nicolsg@tcd.ie)

**Primary supervisor:** Dr Catherine Darker, Trinity College Dublin.

Tel: 01-8968510 Email: [Catherine.darker@tcd.ie](mailto:Catherine.darker@tcd.ie)

**Secondary Supervisor:** Dr Catherine Hayes, Trinity College Dublin.

Tel: 01-8961385 Email: [hayesc9@tcd.ie](mailto:hayesc9@tcd.ie)

If you would like any more information about the study or if you have any further questions, please refer to the attached participant information leaflet or contact the research team.

**Please initial each box to confirm that you have read, understood and agreed to each of the points of the form.**

1. I confirm that I have read and understood the attached Participant Information Leaflet. I have had the opportunity

to think about the information and to ask questions. The research team has answered any questions that I have had.

1. I agree to take part in the research study.
2. I agree that my personal details will not be shared with anyone outside of the research team. A

professional transcriber will have access to the focus group data I provide, and will sign a legally binding

Data Processing Contract governing the data processing as outlined in Article 28 of the General Data Protection Regulation (GDPR). I understand that my data will be anonymised i.e. my name or personal details will not appear, prior to any publication of the results. I understand that I can get a copy of the Privacy Notice from the researcher if I want to find out more about how my data are protected.

1. I agree to my data being stored securely for ten years after the study ends, by Trinity College Dublin researchers.
2. I understand that my participation is voluntary and that I am free to withdraw at any time without giving any

reason. I understand that if I withdraw from the study, any data collected from me can still be used unless I state otherwise. I understand if I need to make a complaint, I can contact the Data Protection Officer at [dataprotectionofficer@tcd.ie](mailto:dataprotectionofficer@tcd.ie).

1. I agree to being part of a pilot study and understand that survey information that I provide will be

confidential to the research team and the focus group will be audio-recorded and put into writing word

for word by a transcriber, and the voice recording will then be destroyed.

__________________________ __________________ ___________________________

(Please print name of participant here) Date (Please sign here)

__________________________ __________________ _________________________

Researcher’s name Date Researcher’s signature
